# Alcohol Health Services Research: An Evolving Agenda

**Published:** 1995

**Authors:** Constance Weisner

**Affiliations:** Constance Weisner, Dr.P.H., is a senior scientist at the Alcohol Research Group, Western Consortium for Public Health, and an adjunct associate professor at the School of Public Health, University of California at Berkeley, Berkeley, California

**Keywords:** health services research, AOD dependence, health care administration, health care costs, health care financing, health care access, health care delivery, treatment outcome, treatment factors, collaboration, AOD prevention

## Abstract

Alcohol health services research analyzes factors affecting the delivery of alcohol-related health services to clients in actual treatment settings rather than under clinically controlled conditions. In recent years, alcohol services research has become an important focus of the National Institute on Alcohol Abuse and Alcoholism’s research agenda.

In 1992 the Alcohol, Drug Abuse, and Mental Health Administration (ADAMHA) Reorganization Act moved the National Institute on Alcohol Abuse and Alcoholism (NIAAA), the National Institute on Drug Abuse, and the National Institute of Mental Health from ADAMHA to the National Institutes of Health. The law also required that the three Institutes allocate at least 15 percent of their budgets to health services research. This mandate focused new attention on health services research as an important area of investigation in the delivery of health services in general and in the treatment and prevention of alcohol and other drug (AOD) abuse and mental health problems in particular. The mandate also has provided the Institutes with an opportunity to expand and better coordinate their efforts in this area.

This article defines the general scope of health services research. It presents some of the influences that have affected the delivery of alcoholism treatment services over the past 20 years and that have implications for health services research in the alcohol field. The article also lists pressing questions for future alcohol-related health services research.

## What Is Health Services Research?

In a definition applicable for all areas of health services, the ADAMHA Reorganization Act (section 409) states that health services research encompasses “. . . research endeavors that study the impact of the organization, financing, and management of health services on the quality, cost, access to, and outcomes of care” (Gordis 1993, p. 1). In other words, this type of research does not address the clinical efficacy of specific treatment approaches but instead examines the provision of health services in “real world” settings and analyzes factors, such as service delivery organization or financing issues, that affect the provision of health services. This broad definition considers the individual patient, the organizations that provide treatment and prevention services, and the wider environment of the community and its institutions, emphasizing connections and referrals in the treatment or management of health problems within and among each of these components.

By doing so, health services research fosters a dynamic interaction among researchers, practitioners, treatment program administrators, and health policy-makers—groups that traditionally maintain different perspectives on health services. For example, researchers typically focus on the existing gaps in knowledge regarding the antecedents, correlates, and effects of disorders, such as alcohol dependence, and treatment efficacy. Consequently, researchers often advocate only carefully studied, incremental developments in prevention, treatment, and policymaking. In contrast, the practitioners’ priority is to treat patients; therefore, practitioners demand practical, effective, readily available tools, such as therapeutic procedures or medications, to help their clients. Administrators and policymakers must make programmatic and fiscal decisions and are subject to demands for cost containment and to increased scrutiny from legislators and board members. To justify administrative decisions and develop new policy directions, administrators and policymakers need “bottom line” information on the effectiveness and efficiency of existing treatment and prevention approaches.

Health services research attempts to integrate these diverse needs and perspectives. It employs scientific research methods to examine treatment and prevention approaches in the real world settings of practitioners. Administrators and policymakers can apply the findings of such studies to larger policy and financing issues. In addition, the results of health services research can inform third-party payers of health services (i.e., insurers) and other community agencies with an interest in health services, such as the criminal justice system or the welfare system.

Although the mandate for health services research is relatively recent, initiatives for this kind of research began more than two decades ago. Already in 1974 Congress established the National Center for Health Services Research as part of the Public Health Service, illustrating the Government’s interest in such research (Wallen 1988). In 1979 the Institute of Medicine (IOM) of the National Academy of Sciences conducted a study focusing on health services research that helped define different aspects of the field (IOM 1979). And in 1983 the Association for Health Services Research (AHSR) was founded as a professional organization advocating health services research (Wallen 1988).

### Levels of Health Services Research

The 1979 IOM report described four levels of problems that can be addressed by health services research (Wallen 1988):

- At the clinical level, health services research studies can assess nonmedical factors influencing treatment outcome (e.g., whether similar treatment in an inpatient or outpatient setting affects the outcome of alcoholism treatment) and address outcome criteria, such as treatment costs, that often are not included in controlled clinical studies.
- Studies at the institutional level analyze the organizational and administrative structure of treatment (e.g., whether treatment is provided by a facility within a provider network or is commissioned to an outside program) and how these factors relate to patient characteristics, access, or treatment outcome.
- At the systems level, health services research studies the interrelationship between different facets of the health care system. For example, researchers could examine whether it is more effective to treat criminal justice clients within the jail setting or to refer them to special treatment facilities.
- Health services research at the environmental level addresses the larger social, political, and economic contexts that affect utilization of health care services, such as public opinions about alcohol consumption or legislative mandates for insurance coverage of alcoholism treatment.

To address these very diverse issues, health services research draws on researchers, methods, and theories from a variety of fields. These include epidemiology, medicine, sociology, psychology, public health, economics, and business administration.

## Health Services Research in the Alcohol Field

The NIAAA report to Congress on health services research (Gordis 1993) described the goal of its alcohol services research program as the development of a knowledge base that can be used to “improve the quality, efficiency, and effectiveness of services for alcohol-related problems” (p. 1). To this end, alcohol health services research addresses questions such as the following: How effective are treatment and prevention interventions in real world settings? And how do the organization and financing mechanisms of alcohol services affect the quality and effectiveness of these services?

Although NIAAA’s alcohol services research received a boost from the mandate in the ADAMHA Reorganization Act, NIAAA already had supported a large body of clinical, epidemiological, and prevention studies falling under the heading of health services research prior to 1992. For example, in the ongoing program of clinical trials research and medication development, researchers not only establish the efficacy of new interventions in randomized clinical trials under controlled conditions but also determine their effectiveness when applied in actual treatment settings. Other examples of alcohol-related health services research have included studies to determine the need for alcoholism treatment in the general population and analyses of the effectiveness of prevention interventions.

Alcohol services research encourages long overdue partnerships and cooperation between the research, treatment, and prevention communities in the alcohol field and with the public at large. For example, as a consequence of federally mandated support of alcohol services research, health services researchers, AHSR, and several private foundations have begun to work jointly toward developing a comprehensive research agenda and to narrow the long-criticized gap between researchers and practitioners (Gordis 1991; Woody et al. 1991). Recent examples of these efforts include newsletters that communicate research findings and provide other links between research and practice, such as *Frontlines*, which is cosponsored by the Foundation for Health Services Research and NIAAA, and *Science Matters*, which is sponsored by the Johnson Institute Foundation. Private foundations, such as the Robert Wood Johnson Foundation, also emphasize aspects of alcoholism prevention and treatment services research in their projects.

## The Political and Social Context for Alcohol Services Research

In the 25 years since NIAAA was established, substantial changes have occurred in the social environment of the alcoholism treatment system. These include expanded treatment capacities, particularly in the private sector, combined with efforts toward cost containment; decentralized public treatment administration; merging of alcoholism and drug abuse treatment agencies; increasing connections between different community agencies dealing with clients with alcohol problems; changing public opinion about alcohol problems; a growing diversity of clients; and research efforts to improve the matching of patient characteristics to appropriate treatment approaches. Many of these developments have helped shape the alcohol services research agenda during the 1980’s and 1990’s. The implications of these and other changes have not been assessed fully and therefore will be important topics of health services research in the coming years.

### Increased Insurance Coverage and Treatment Capacities

Perhaps most importantly for the alcohol field, advocacy by NIAAA and its constituent groups resulted in a widespread increase in insurance coverage for alcohol-related problems by the late 1980’s, when most States mandated that individuals be given the option for such coverage (Roman 1988; IOM 1990). One result of this development was a considerable increase in alcoholism treatment capacity. The total number of alcoholism-only and combined AOD abuse treatment facilities increased from 4,233 in 1982 to 7,766 in 1990 (Schmidt and Weisner 1993).

**Figure 1:**
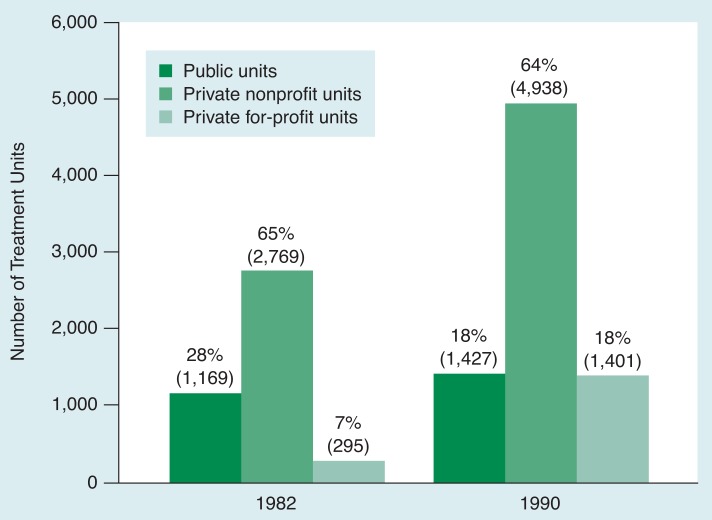
The number and ownership of alcoholism treatment units in 1982 and 1990. The data were obtained from the 1982 and 1990 National Drug and Alcoholism Treatment Unit Surveys. The units listed include alcohol-only and combined alcohol and other drug abuse treatment units. The percentages refer to the proportion of the unit types in the respective survey years.

The growth in alcoholism treatment capacity has not affected the public and private treatment sectors and all geographical areas equally. For example, financing policies helped create a two-tiered system of public programs emphasizing outpatient services without intensive medical support and private facilities offering a higher proportion of cost-intensive inpatient and medically supported services (Yahr 1988). During the 1980’s the share of public programs decreased from 28 percent to 18 percent of the total units, whereas the percentage of private, for-profit programs increased from 7 percent to 18 percent (Schmidt and Weisner 1993). Private, not-for-profit facilities consistently represented approximately two-thirds of all units, and their funding and client numbers increased proportionally during that time (figure 1).

According to an IOM report (1990), the changes in the proportion of public and private treatment capacities do not always match the number of people in need of alcoholism treatment. For example, the private treatment sector rather than the public treatment sector might be expanded in a geographical area, even though public programs might be more appropriate and needed for the area’s client population. Instead, factors related to funding mechanisms often influence treatment provision in specific geographical regions. Alcohol services research has yet to address the underlying relationships between need and actual provision and utilization of treatment services.

Although the range of private treatment programs expanded dramatically during the 1980’s, these programs only rarely were included in treatment outcome research (IOM 1990). Fragmented and unmonitored funding through many types of insurance plans, as well as the lack of overall policy and regulation of the private sector, created few incentives or mandates for private programs to participate in outcome research. Thus, researchers may have missed the opportunity to study the effectiveness of the programs’ characteristics, such as longer lengths of stay for specific client subgroups (for an example, see Nace 1993).

#### Managed Care

The most notable development related to insurance coverage for alcoholism treatment, in addition to the increase in its availability, has been the growing use of managed care organizations (e.g., health maintenance organizations or preferred provider organizations). These groups are designed to contain health care costs through mechanisms such as emphasizing prevention and early care, restricting unnecessary treatment, reducing the average length of care, emphasizing outpatient treatment settings, reducing hospital-based treatment approaches, or instituting case-monitoring strategies. Although managed care arrangements so far primarily have involved private facilities, State and county treatment systems and public insurance, most notably medicaid, increasingly have adopted similar cost-containment mechanisms (Rogowski 1992; Levin et al. 1984). On the whole, these changes represent a shift in the control over decisions of patient care from the provider to the payer. However, little is known about the coordination of alcoholism treatment with primary care services or the effectiveness of program organizations in managed care structures.

### Government Deregulation

Until 1982 NIAAA administered all Federal funding to the States for alcoholism treatment and prevention programs. After 1982, however, the Federal Government provided funding for these programs directly to the States in the form of relatively unrestricted alcohol, drug, and mental health block grants. This change initiated an era of Government deregulation, in which the requirement of programs receiving public funds to collect data on clients and their treatment outcome was reduced significantly. Thus, at the same time that alcoholism treatment capacities expanded as a consequence of greater insurance coverage, data collection on the clients was relaxed. As a result, alcohol services researchers have a difficult task tracking how the dramatic changes in treatment delivery (e.g., increased availability, increased insurance coverage, or changing funding mechanisms) affected the characteristics of clients in the system and treatment efficacy.

### Combination of Alcoholism and Other Drug Abuse Treatment

Another far-reaching change concerning alcoholism treatment during the 1980’s was the merging of alcoholism and other drug abuse treatment and prevention programs at the State and local levels (Schmidt and Weisner 1993). For example, more than 65 percent of the 7,759 public and private programs analyzed in the 1989 National Drug and Alcoholism Treatment Unit Survey (NDATUS) reported as combined AOD abuse programs an increase of 234 percent compared with the corresponding 1982 survey (Schmidt and Weisner 1993). In contrast, alcohol-only and drug-only facilities decreased by 46 percent and 16 percent, respectively (Schmidt and Weisner 1993). The establishment of the Office for Substance Abuse Prevention in 1986 and the Office for Treatment Improvement in 1989 (now the Center for Substance Abuse Prevention and the Center for Substance Abuse Treatment, respectively) also symbolized the focus on combined AOD abuse treatment.

At least in part, the merger of AOD abuse treatment agencies reflects the growing proportion of individuals in the general population and in the treatment population who abuse more than one drug (Schmidt and Weisner 1993). However, it is important to note that the special prevention and treatment needs of multiple drug abusers have not been researched adequately, and treatment models for this population have not been developed and evaluated (Hubbard 1990). Consequently, the prevention and treatment of multiple drug abuse offer a wide open field for health services research.

### Diversity of Agencies Working With People With Alcohol Problems

**Figure 2:**
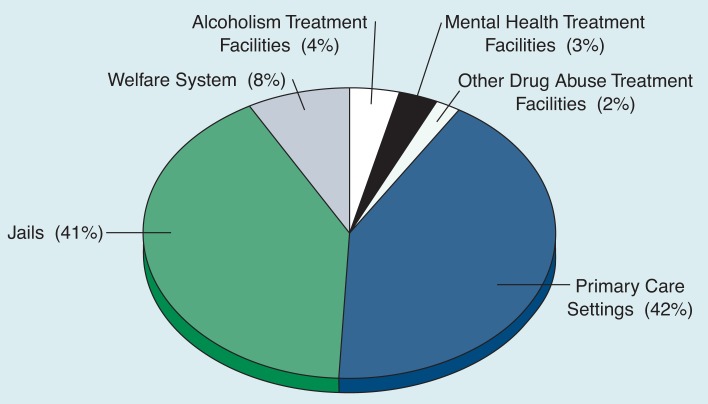
Distribution of problem drinkers in health and community agencies in one California county. The term “problem drinker” is used as defined by the Institute of Medicine (1990). Problem drinkers were identified by surveying all clients in these agencies using health questionnaires. SOURCE: Weisner 1993.

Health surveys among the clients of various community agencies have shown that many people with alcohol problems do not receive treatment in specialized settings. Instead, these people are found in a wide range of nonspecialized health and social service settings within the community, such as emergency rooms, primary health care practices, other drug abuse and mental health treatment services, or welfare and criminal justice agencies (Regier et al. 1993; Weisner and Schmidt 1992). For example, a survey of health and community agencies in one California county showed (figure 2) that only 4 percent of all problem drinkers^1^ were in alcoholism treatment facilities, 3 percent were in mental health facilities, and 2 percent were in other drug abuse treatment facilities. In contrast, 42 percent of the problem drinkers were identified in primary care settings, 41 percent in jails, and 8 percent through the welfare system (Weisner 1993). Although only reflecting the distribution of problem drinkers in one geographical area, these numbers illustrate the diversity of settings in which individuals with alcohol problems are found.

Because of the wide distribution of clients with alcohol problems, an increasing need exists for health services research to analyze how effectively and efficiently the various agencies involved respond to these clients and their needs. This need for research is reinforced by the increasing numbers and types of problems attributed to or associated with alcohol abuse and dependence, such as family violence, problems in the workplace, alcohol-induced neonatal health problems, and HIV infection and AIDS.

#### Mandated Treatment

As mentioned above, a large proportion of people with alcohol problems are identified in the criminal justice system and subsequently are coerced into treatment. For example, large numbers of drinking and driving offenders are referred to alcoholism treatment as an alternative or added sanction to jail sentences or fines. Similarly, employee assistance programs (EAP’s) increasingly require employees with alcohol problems to attend treatment. Consequently, many programs now serve a high proportion of coerced clients (IOM 1990). This trend could have important implications for overall treatment access (e.g., limited availability to clients entering treatment voluntarily) and outcome (because coerced clients’ motivation levels may differ from those of voluntary clients). However, alcohol services research has yet to address these issues comprehensively.

### Changes in Public Opinion

Changes in public opinion about alcohol consumption and alcoholism also have affected alcoholism treatment and treatment policies. These changes have included a trend toward decreased per capita alcohol consumption in the population, increased concern about drinking and alcohol-related problems, and increased use of treatment services (Weisner et al. 1995). Organized groups, such as Mothers Against Drunk Driving, have contributed to these developments by mobilizing public concerns and influencing political opinions about alcohol problems. Ongoing advocacy by prominent and vocal opinion leaders who are recovering alcoholics (e.g., former First Lady Betty Ford) also has positively affected the public’s attitudes toward prevention and treatment. However, alcohol services research studies still are needed to assess the growth and impact of these social forces.

### Changes in Patient Populations

The characteristics of treatment delivery as well as the epidemiological characteristics of the treatment population have changed over the past two decades. For example, as mentioned earlier, increasing numbers of clients with combined AOD problems are entering the treatment system (Schmidt and Weisner 1993). Researchers and practitioners have noted an increased prevalence of psychiatric co-morbidity among patients in alcoholism treatment (Helzer and Pryzbeck 1988). Programs designed to promote early detection of alcohol problems in the workplace (e.g., EAP’s) and other settings have identified clients who often are younger and who exhibit less severe symptoms (Schmidt and Weisner 1993). Policy developments at the national level, such as increased funding for programs targeted at women and, more specifically, pregnant women, have resulted in growing numbers of female patients (Schmidt and Weisner 1993). All these trends have resulted in a more heterogeneous treatment population.

Coinciding with the patient population diversification, treatment approaches also have become more varied. For example, the NDATUS surveys found an increasingly diverse treatment system with larger numbers of self-help groups, early intervention programs, and programs for drinking and driving offenders (Schmidt and Weisner 1993). The effects of this heterogeneity of patients and programs on treatment outcome and the effectiveness of specialized programs rarely have been evaluated and should be addressed by health services researchers.

### Increased Focus on Patient-Treatment Matching

Researchers and practitioners increasingly have realized that not all individuals with alcohol problems respond equally to all treatment approaches. Consequently, research regarding the matching of specific client characteristics to specific treatment service characteristics has been increasing during the last decade. This research has included both individual research projects and larger coordinated trials, such as the NIAAA-sponsored Project MATCH (U.S. Department of Health and Human Services 1994). However, health services research describing how the findings of such controlled studies can be generalized to actual treatment settings is lacking.

## Future Questions for Alcohol Services Research

The developments described above point to several important questions that need to be considered in alcohol services research in the coming years. Many of these questions relate to the current emphasis on developing new health care policies.

- What are the effects of cost-containment efforts (e.g., managed care organizations, the focus on outpatient services, or decreased lengths of stay in inpatient treatment) on treatment access, quality, and effectiveness? This particular issue also includes the questions of whether treatment effectiveness meets the expectations of health and social policymakers and payers (i.e., Is treatment effective enough to warrant the costs incurred?) and which modalities are cost-effective for which patient population.
- What costs associated with alcoholism treatment should be included in health care coverage? And what are the advantages and disadvantages of integrating both public and private alcoholism services into one overall health care delivery system versus maintaining a separate public treatment system? These questions are important because clients who traditionally use public services have very different treatment needs (e.g., needs for ancillary services, such as housing or vocational training) from clients in private facilities.
- What is the relationship between need and demand for alcoholism services? Determining how many people with alcohol problems actually would enter treatment if coverage of alcoholism treatment was provided for everyone is complex, because one must consider the effects of diverse factors, such as developments in social policies or client coercion, on treatment utilization.
- What is the impact of different financing mechanisms—for example, reimbursing the treatment provider only up to a predetermined, fixed amount per patient—for alcoholism prevention and treatment services, both in the public and private sector, on service delivery (i.e., treatment practices, modalities, or length of stay)? Similarly, how do various reimbursement and organizational approaches (e.g., managed care) affect treatment cost, access, and outcome?
- What is the effect of combining AOD abuse treatment services on coverage, access, content, and effectiveness of alcoholism treatment?

In addition to answering these questions, alcohol services research could contribute to a more accurate description of client characteristics in public and private alcoholism treatment facilities. Such information is needed to enhance the understanding of national treatment needs and of the structure of national prevention and treatment service systems. Some long-term studies of the general population (e.g., the National Health Interview Survey and the National Alcohol Survey) already exist, but a comparable comprehensive survey of the treatment population still needs to be conducted.

## Obstacles in Alcohol Services Research

Alcohol services research, like any kind of health services research, is faced with an inherent tension between the different priorities and demands of policymakers, service providers, and scientists. This tension is reflected in the difficult conceptual issues that arise when trying to formulate specific health services research questions. For example, how are the questions under study defined—based on the priorities of the payer (e.g., cost containment for the health care organizations) or the treatment provider (e.g., outcome for the individual client)?

When designing rigorous scientific studies in actual treatment settings, researchers often face logistical difficulties. For example, many questions addressing the cost-effectiveness of specific treatment approaches can be answered most rigorously through controlled trials of different modalities within one treatment agency. However, it can be exceedingly difficult to implement such studies within ongoing programs in which the priorities are the benefit to and continued care of the clients, which may be at odds with the methodology required for a scientific study. Valid health services research studies require an elaborate study design, the collaboration of the program staff in the strict implementation of the study protocol, and mechanisms to integrate intensive scientific assessment into normal treatment protocols. And in many cases, all these demands must be met in the context of limited financial resources.

## Mechanisms to Facilitate Alcohol Services Research

The NIAAA National Advisory Council’s Subcommittee on Health Services Research and other interested groups, such as AHSR, have identified several mechanisms to improve and facilitate alcohol services research (Weisner 1993). Research review groups of experts could evaluate the special opportunities, limitations, and methodological approaches necessary for effective health services research studies. Also, the secondary analyses^2^ of existing data sets established for other purposes, such as general population surveys or insurance claims data, may provide valuable material for alcohol services research and therefore warrant more attention and funding.

Sufficient funding also is a crucial factor in facilitating alcohol services research. Studies that meet the requirements of rigorous scientific standards within the complex and demanding environments of the general population and real-world prevention and treatment settings by their nature often require larger costs per grant. For example, research on minorities and other priority populations that are important objects of health services research often cannot be examined without significant funds because of the large sample sizes required and/or the diverse range of agencies and communities involved.

## Summary

Alcohol services research strives to evaluate the many different factors influencing the delivery of alcoholism treatment to patients in actual treatment settings rather than under controlled clinical conditions. To reach this goal, researchers, practitioners, and policymakers must collaborate closely. This cooperation may provide substantial benefits to the alcohol field. Institutional ties and partnerships among these groups and with new constituency groups may help to communicate findings and provide advocacy for the full range of health services research in the alcohol field. In addition, alcohol services research can attract new researchers who have new perspectives that may enrich the existing disciplinary approaches with methodological and other innovations, thus invigorating the entire alcohol field.

## Footnotes

1 In this survey, the classification of “problem drinker” is based on the level of alcohol consumption, the social consequences of drinking, and the presence of dependence symptoms (IOM 1990).

2 A secondary analysis is a reanalysis of previously accumulated data from a different angle or with regard to new questions.
